# A High-Throughput Sequencing Data-Based Classifier Reveals the Metabolic Heterogeneity of Hepatocellular Carcinoma

**DOI:** 10.3390/cancers15030592

**Published:** 2023-01-18

**Authors:** Maolin Ye, Xuewei Li, Lirong Chen, Shaocong Mo, Jie Liu, Tiansheng Huang, Feifei Luo, Jun Zhang

**Affiliations:** 1 Department of Digestive Diseases of Huashan Hospital, Institutes of Biomedical Sciences, Fudan University, Shanghai 200040, China; 2 Shanghai Guanghua Hospital of Integrated Traditional Chinese and Western Medicine, Shanghai 200052, China

**Keywords:** metabolic heterogeneity, hepatocellular carcinoma, tumor microenvironment, metabolic competition

## Abstract

**Simple Summary:**

The metabolic heterogeneity complicates the clinical treatment of hepatocellular carcinoma. In this study, we classified hepatocellular carcinoma into two clusters based on their energy metabolic pathways’ activities. We found this classification system correlated with several clinical characteristics and molecular profiles of liver cancer patients. We also proposed and validated targeted metabolic therapy by exploiting human liver cancer cell lines. Additionally, we revealed that cancer cells might impair the anti-tumor function of cytotoxic T cells through metabolic competition.

**Abstract:**

Metabolic heterogeneity plays a key role in poor outcomes in malignant tumors, but its role in hepatocellular carcinoma (HCC) remains largely unknown. In the present study, we aim to disentangle the metabolic heterogeneity features of HCC by developing a classification system based on metabolism pathway activities in high-throughput sequencing datasets. As a result, HCC samples were classified into two distinct clusters: cluster 1 showed high levels of glycolysis and pentose phosphate pathway activity, while cluster 2 exhibited high fatty acid oxidation and glutaminolysis status. This metabolic reprogramming-based classifier was found to be highly correlated with several clinical variables, including overall survival, prognosis, TNM stage, and 𝛼-fetoprotein (AFP) expression. Of note, activated oncogenic pathways, a higher TP53 mutation rate, and increased stemness were also observed in cluster 1, indicating a causal relationship between metabolic reprogramming and carcinogenesis. Subsequently, distinct metabolism-targeted therapeutic strategies were proven in human HCC cell lines, which exhibit the same metabolic properties as corresponding patient samples based on this classification system. Furthermore, the metabolic patterns and effects of different types of cells in the tumor immune microenvironment were explored by referring to both bulk and single-cell data. It was found that malignant cells had the highest overall metabolic activities, which may impair the anti-tumor capacity of CD8+ T cells through metabolic competition, and this provided a potential explanation for why immunosuppressive cells had higher overall metabolic activities than those with anti-tumor functions. Collectively, this study established an HCC classification system based on the gene expression of energy metabolism pathways. Its prognostic and therapeutic value may provide novel insights into personalized clinical practice in patients with metabolic heterogeneity.

## 1. Introduction

Liver cancer has become one of the leading causes of cancer-related deaths worldwide [1], with approximately 0.9 million new cases and 0.8 million deaths every year [2]. Primary liver cancer includes two main subtypes: hepatocellular carcinoma (HCC) and intrahepatic cholangiocarcinoma. Although HCC accounts for 75–85 percent of all liver cancer cases [2], the molecular classification of HCC is not well established due to its heterogeneous nature. The Barcelona Clinic Liver Cancer Classification is the primary classification method in clinical practice, but it only reflects the clinical status of HCC patients [3]. Additionally, no revolutionary anticancer agents or therapies for liver cancer have been reported over the past 30 years [4]. In recent years, rapidly advancing omics techniques have revealed a landscape of molecular alterations in HCC that are associated with tumor progression [5]. These make the molecular classification of HCC extremely promising and urgent, which would shed light on personalized medicine targeting molecular alterations in HCC.

Metabolic reprogramming has long been recognized as one of the hallmarks of cancer [6], which fuels the proliferation and metastasis of cancer cells [7]. Cancer cells share the same phenotype of sufficient energy generation and metabolite conversion required for unrestricted proliferation in a relatively nutrient-poor microenvironment. They also obtain known metabolic changes, including the deregulated uptake of glucose and amino acids, unrestricted biosynthesis using metabolic intermediates, etc. [8]. Alterations in glucose, amino acid, and lipid metabolism are highly prevalent in HCC, regardless of the etiology. Efforts have been made to exploit the metabolic reprogramming and to search for biomarkers and therapies for HCC [9]. Reducing glutaminolysis using the glutaminase inhibitor CB-839 impaired the viability of several human HCC cell lines and improved the prognosis of corresponding xenograft mouse models [10]. However, the therapeutic efficacy has not reached satisfactory levels in the last decade due to metabolic heterogeneity [9], which must be further elucidated.

Recently, Gao et al. classified hepatitis B virus-associated HCC patients into different metabolic subtypes based on their proteogenomic metabolism profiles [11]. Another study constructed a classifier based on the transcriptional expression of 2752 metabolically related genes and divided HCC patients into metabolic high, metabolic intermediate, and metabolic excluded subtypes [12]. Bidkhori et al. [13] subdivided HCC patients into different clusters with distinct metabolic networks by exploiting multiomics datasets. These studies indicate the possibility of classifying HCC according to different metabolic patterns. However, these studies used the overall metabolic status to classify HCC in a way that neglected the differences among different metabolic pathways. In addition, efforts have been made to investigate the prognosis of liver cancers with individual metabolic pathways, like the glucose, lipid, and asparagine metabolic pathways [14,15,16]. Previous studies have shown that deregulated metabolic pathways and their corresponding intermediates directly change cell signaling in liver cancer [17], while alterations in different pathways confer distinct characteristics on cells [18]. These findings provide the basis for proposing a new classification standard for HCC based on the activity levels of different metabolic pathways.

In this study, several independent cohorts were used to robustly identify two subtypes of HCC metabolism based on activity levels of four major energy metabolism pathways, including glycolysis, the phosphate pentose pathway, fatty acid oxidation, and glutaminolysis [18,19]. Subsequently, the prognosis values, clinical characteristics, oncogenic pathway activities, somatic mutation patterns, and potential metabolic therapies of the HCC subtypes were compared. In addition, the constitution and crosstalk of different subclasses of cells in the tumor immune microenvironment were also analyzed via the metabolic view from both bulk and single cell data. The main differences between our study and the research mentioned above are as follows: (1) we proposed targeted therapy focusing on energy metabolic-related pathways and validated the possibility by exploiting hepatocellular carcinoma cell lines, and (2) metabolic competition between malignant cells and anti-tumor immune cells was revealed by using both bulk and single-cell high-throughput sequencing data.

## 2. Materials and Methods

### 2.1. Data Acquisition and Preparation

The Cancer Genome Atlas (TCGA, https://portal.gdc.cancer.gov/ (accessed on 19 April 2022)) and the International Cancer Genome Consortium (ICGC, https://dcc.icgc.org/ (accessed on 1 May 2022)) databases were used to obtain bulk RNA-seq data and clinical information in liver hepatocellular carcinoma (LIHC). Only patients who had complete survival information were kept for additional evaluation. TCGA-LIHC cohorts [20] were used for analysis, and ICGC-LIHC cohorts [21] were used for external validation. Gene expression data from GSE190928 [22], GSE172318 [23], and GSE125449 [24] were retrieved from the Gene Expression Omnibus (GEO, https://www.ncbi.nlm.nih.gov/geo (accessed on 1 May 2022)). Four energy metabolic pathway activities of each cell in GEO: GSE125449 were calculated using the R package Seurat. CD8^+^ T cells were classified into four subtypes based on previously reported markers [25,26].

### 2.2. Classification of Liver Cancer Metabolic Subtypes

Unique feature gene panels for each energy metabolic pathway were obtained from the latest literature [19] and the Kyoto Encyclopedia of Genes and Genomes (KEGG) website (https://www.genome.jp/kegg/ (accessed on 1 May 2022)). Through transcriptional data, we calculated the corresponding energy metabolic pathway scores for each liver cancer sample using single-sample gene set enrichment analysis (ssGSEA) [27]. Following that, we classified liver cancer patients into distinct clusters using unsupervised consensus clustering. Additionally, the optimal cluster number of this classification system was determined by the Probably Approximately Correct (PAC) algorithm. 

### 2.3. Prognostic Analysis of Metabolic Subtypes

Kaplan–Meier survival curves were generated using the R package Survminer. In order to reveal the prognostic value of metabolic patterns, we used both univariate and multivariate Cox proportional hazards models. Tumor weight, metabolic subtypes, and 7 other clinical features were analyzed using the univariate Cox proportional hazards model. Then, five significant variables (*p* value < 0.01) were analyzed in the multivariate Cox proportional hazards model. Forest plots were drawn to show the hazard ratios (HR) and 95% confidential intervals (CI) of each covariate. Further validation of the metabolic classifier was conducted by generating time-dependent receiver operating characteristic (ROC) curves and calculating their area under the curves (AUC). Additionally, the R package pRRophetic [28] was used to estimate the sorafenib sensitivity of HCC patients and liver cancer cell lines.

### 2.4. Estimation of Immune Cell Infiltration

We used the ESTIMATE [27] algorithm to calculate the immune and stromal scores of each sample. Then MCP-counter [29] analysis was conducted to estimate the infiltration of 8 immune and 2 stromal cell populations. Tumor immune dysfunction and exclusion (TIDE) [30], a computational algorithm that can identify factors underlying the mechanisms of tumor immune escape, was performed to estimate the potential immune checkpoint blockade (ICB) response.

### 2.5. Differential Gene Expression Analysis and Pathway Enrichment Analysis

We used the DESeq2 [31] package to conduct differential gene expression analysis for two metabolic subtypes in R. The significance threshold for defining DEGs was adjusted to a *p* value < 0.05 with an absolute log fold change (FC) greater than 1. Then gene set variation analysis (GSVA) [27] was applied to calculate the relative pathway enrichment for two clusters. We used the Molecular Signature Database (MSigDB) [32] to obtain the hallmark gene sets (version 7.5.1) used in this analysis. The R package limma [33] was used to determine the difference in pathway enrichment.

### 2.6. Comparison of Oncogenic Pathway Activity

Ten oncogenic pathway gene sets were obtained from previously published articles [34]. A total of 331 genes in the oncogenic pathways were divided into activated genes and repressed genes according to their corresponding gene functions. The ssGSEA algorithm was used to calculate the enrichment scores of corresponding metabolic pathways in each liver cancer sample. Additionally, activated scores were subtracted from repressed scores to calculate activity scores.

### 2.7. Somatic Mutation Patterns

The somatic mutation data of TCGA-LIHC patients as a MAF file generated using the MuTect pipeline were downloaded from the TCGA database in order to compare the somatic mutation patterns between the distinct clusters. The R package Maftools was used to analyze and visualize the mutation data [35].

### 2.8. Cancer Stemness Index (CSI)

The cancer stemness index algorithm was developed in a previously published article to assess the degree of cancer cell dedifferentiation [36]. Transcription data of human stem cells were downloaded from the Progenitor Cell Biology Consortium (PCBC) (https://www.synapse.org (accessed on 1 May 2022)). The one-class logistic regression (OCLR) machine-learning algorithm was used to construct the stemness model. The CSI of each sample was then calculated using this model.

### 2.9. Human Liver Cancer Cell Lines and Cell Proliferation Assay

Human liver cancer cell lines SNU-449 and Hep G2 were obtained from the Type Culture Collection of the Chinese Academy of Sciences (Shanghai, China). All cancer cells were cultured following the American Type Culture Collection (ATCC) instructions. The proliferation rates of two cell lines were measured using Cell Counting Kit-8 (CCK-8) (Dojindo, Mashiki, Japan). Briefly, 2000 cells per well were seeded in 96-well plates (Corning, New York, NY, USA.). After incubation for 24 h, four metabolic inhibitors, purchased from Selleckchem, were added to each well. After 48 h, CCK-8 solution was added to the cells, and the optical density (OD) values were measured at 450 nm after 60 min incubation. 

### 2.10. Lactate Production Measurement

The lactate production of cells was measured using a lactate assay kit (Sangon Biotech, Shanghai, China). Cells were seeded in the 6-well plate in triplicates. Supernatants were collected after 24 h incubation. The lactate level was normalized to the cell numbers. 

### 2.11. Cell Cycle Analysis

Cell cycle analysis was conducted following the manufacturer’s instructions. Briefly, SNU-449 and Hep G2 were seeded in 48-well plates. Both 2DG and Etomoxir were added to each well, and cells were harvested after incubation for 24 h. The supernatant was removed by centrifugation at 500 g for 5 min. Then, the cells were fixed by adding 1 mL of 75% ethanol overnight. Following that, cells were incubated for 30 min at room temperature with the Cell Cycle and Apoptosis Analysis Kit (Beyotime Biotechnology) after being washed twice using 1 mL of PBS. The stained cells were subjected to cell cycle analysis using a flow cytometer (Cytoflex; Beckman Coulter, IN, USA), and the data were analyzed using Flowjo software (version 10.8).

### 2.12. Statistical Analysis

R software version 4.1.2 was applied for statistical analysis. A two-sided Wilcoxon rank-sum test and chi-square test were used to compare the differences in variables between clusters. Survival analysis was conducted using the log-rank test. A value of *p* < 0.05 was used to define statistical significance. 

## 3. Results

### 3.1. Metabolic Pattern Classifies HCC into Two Subtypes with Clinical Significance

**Figure 1:**
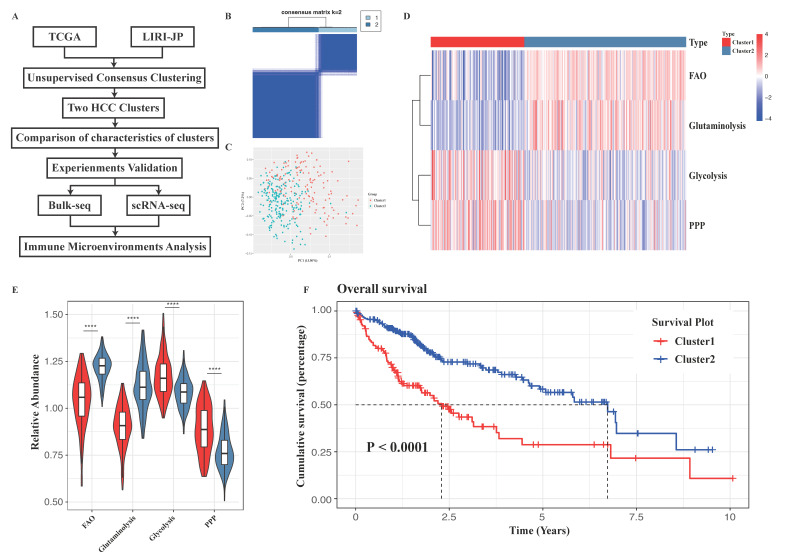
Metabolic heterogeneity of liver cancer is associated with prognosis. (**A**) Schematic diagram of this research. (**B**) A heatmap displaying consensus clustering of metabolic patterns using the k-means algorithm (k = 2). (**C**) Principal component analysis of two metabolic subgroups in the TCGA-LIHC datasets. (**D**) Heatmap displaying the activities of four energy metabolic pathways in liver cancers as calculated by the ssGSEA algorithm. (**E**) Relative energy metabolic pathway activity of two clusters. (**F**) Kaplan–Meier curves of OS between clusters in the TCGA-LIHC datasets. Log-rank test *p* values are shown. PPP, pentose phosphate pathway; FAO, fatty acid oxidation; ****, *p* value < 0.0001.

To reveal the heterogeneity in the energy metabolism patterns of HCC, ssGSEA scores were used to represent the relative metabolic activity of each sample from the TCGA-LIHC database based on four central metabolic pathways: glycolysis, phosphate pentose pathway (PPP), fatty acid oxidation (FAO), and glutaminolysis (Table S1). Figure 1A demonstrates an overview of this study. The unsupervised consensus clustering algorithm was conducted to identify the metabolic subtypes of HCC patients. By using a consensus heatmap (Figure 1B), the PAC algorithm, and principal component analysis (PCA, Figure 1C), it was revealed that the optimal clustering number was two, in which cluster 1 exhibited higher glycolysis and PPP activities while cluster 2 relied more on the other two energy metabolic pathways (Figure 1D,E), which were also validated in the ICGC-LIHC cohort repeatedly and robustly (Supplementary Figure S1A,B).

Kaplan–Meier analysis showed that in both cohorts, the patients in cluster 1 had poorer overall survival (OS) than those in cluster 2 (Figure 1F and Figure S1C). Additionally, several clinical variables were different between the 2 clusters in the TCGA datasets, including AFP value, pathological stage, T stage, and tumor weight (Table 1). Four variables selected by univariate Cox regression analysis were used to create a multivariate Cox proportional hazards regression model, which showed that cluster 1 correlated with a worse prognosis in liver cancer patients (Supplementary Figure S1D). The area under the curve (AUC) of metabolic patterns in the TCGA set for 1, 3, and 5 years was 0.674, 0.642, and 0.644, respectively (Supplementary Figure S1E). The estimated IC50 values of sorafenib were significantly higher in cluster 1, indicating that patients in cluster 1 tend to be more resistant to sorafenib therapy than those in cluster 2 (Supplementary Figure S1F). These results demonstrated the reliability of the energy metabolism classifier and its clinical significance in HCC.

### 3.2. The Differentially Expressed Genes and the Difference in Pathway Enrichment

**Figure 2:**
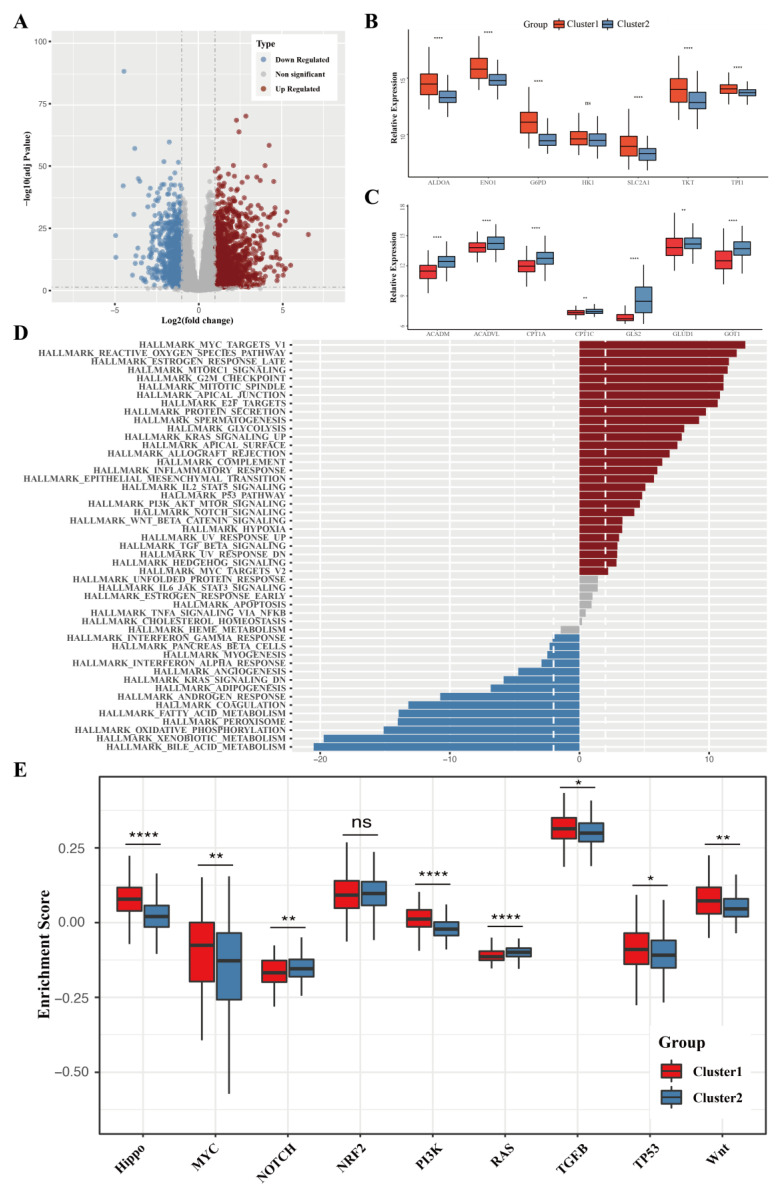
Distinct transcriptomic features between two liver cancer metabolic subtypes. (**A**) A volcano plot displaying differentially expressed genes in two clusters. (**B**,**C**) Relative expression of chosen genes of the glycolyis and PPP (**B**) and chosen genes of the FAO pathway and glutaminolysis (**C**) in the TCGA-LIHC datasets. (**D**) GSVA enrichment analysis of DEGs. (**E**) Quantification of 10 classical oncogenic pathways’ activities in two clusters. ns, *p* value > 0.05; *, *p* value < 0.05; **, *p* value < 0.01; ****, *p* value < 0.0001.

Differential expression analysis was carried out to better characterize the differences between these two HCC subtypes. A total of 1410 genes were shown to be upregulated and 755 genes were downregulated in cluster 1 of the TCGA cohort (Figure 2A). Cluster 1 showed higher expression of glycolysis- and PPP-associated genes, while most genes in the FAO and glutaminolysis pathways were enriched in cluster 2 (Figure 2B,C). A GSVA enrichment analysis was then performed to understand the biological meaning of these differentially expressed genes (Figure 2D). Not surprisingly, HALLMARK GLYCOLYSIS was among the upregulated pathways, and HALLMARK_FATTY_ACID_METABOLISM was among the downregulated pathways in cluster 1. To better evaluate the enrichment of oncogenic pathways, their activity scores were calculated using the ssGSEA algorithm (Figure 2E). Almost all the activity levels of oncogenic pathways were different between these two clusters. Among the ten classical oncogenic pathways, cell cycle-related, hippo, MYC, PI3K, TGF-β, P53, and Wnt pathways had higher activity scores in cluster 1, whereas only Notch and Ras pathways were enriched in cluster 2. The above results were also validated in the ICGC cohort (Supplementary Figure S2A,B). These findings suggest that HCC patients whose energy supply is largely derived from glycolysis and PPP tend to have more malignant behaviors.

### 3.3. Metabolic Status-Specific Somatic Mutation Pattern of HCC

**Figure 3:**
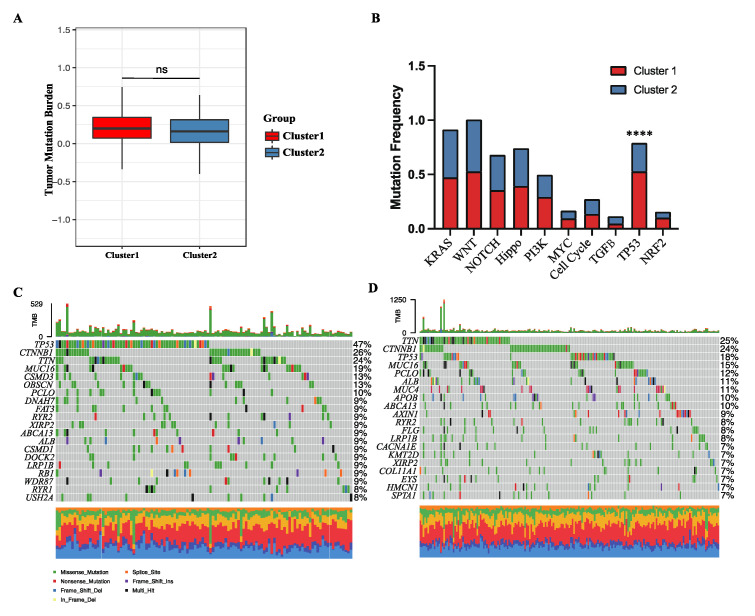
Comparison of the somatic mutation frequency alteration in two clusters. (**A**) Quantification of the tumor mutation burden in two clusters. (**B**) Mutation frequency of 10 classical oncogenic pathways. (**C**,**D**) The 20 genes with the highest mutation frequency in cluster 1 (**C**) and cluster 2 (**D**) in OncoPrint. ns, *p* value > 0.05; ****, *p* value < 0.0001.

Oncogene mutation plays a key role in the progress of carcinogenesis. To shed light on the effect of somatic mutation patterns on the activity of oncogenic pathways, somatic mutation data for HCC patients were obtained from the TCGA-LIHC cohort. Two clusters showed comparable overall tumor mutation burden (TMB) (Figure 3A), while interestingly, among 10 classical oncogenic pathways, only the TP53 pathway had a significantly higher mutation rate in cluster 1 (Figure 3B). As one oncogenic gene alone can significantly impact the prognosis of cancer [37], the difference in mutation frequency of every single gene was further compared between the two clusters using the Fisher’s exact test (Figure 3C,D). Again, TP53 was detected as the only oncogene exhibiting a distinct mutation pattern. These results suggest that the TP53 mutation may at least partially account for the difference in the enrichment of oncogenic pathways between the two clusters. However, given that the activity levels of the other nine oncogenic pathways were significantly different between the two clusters, the effect of energy metabolism reprogramming on the enrichment of oncogenic pathways was more likely to arise at the transcriptional level.

### 3.4. Cancer Stem Cell Index and Its Correlation with Energy Metabolic Pathways

**Figure 4:**
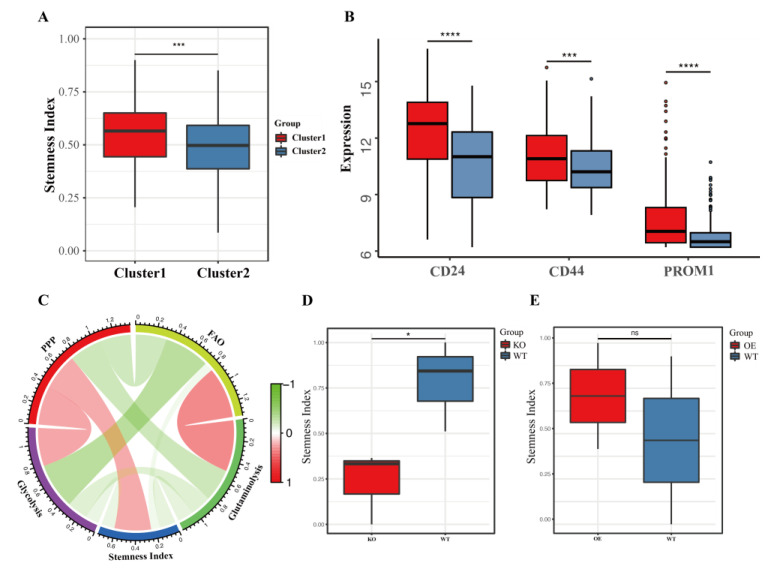
PPP activity regulates the stemness of cancer cells. (**A**) Quantification of the stemness index for two clusters. (**B**) Relative expression of stemness-associated genes. (**C**) A chord diagram displaying the correlation between four energy metabolic pathway activities and liver cancer stemness. (**D**,**E**) Comparison of tumor cell stemness indices after knockout (**D**) or overexpression (**E**). KO, knockout; OE, overexpression. ns, *p* value > 0.05; *, *p* value < 0.05; ***, *p* value < 0.001; ****, *p* value < 0.0001.

A recent study found that human stem cells repeatedly acquire and expand TP53 mutations, which confer a growth advantage during cell culture [38]. Thus, the stemness of each sample was also assessed using the OCLR machine learning algorithm, which calculated the cancer stem cell index (CSI) [36]. It was found that the CSIs were significantly higher in cluster 1 (Figure 4A), which was well consistent with the higher oncogenic pathway activity and more somatic mutations in cluster 1. In addition, the expression levels of several liver cancer stem cell markers [39] were all significantly higher in cluster 1 (Figure 4B). To determine which energy metabolic pathway contributes most to HCC stemness, the correlation between the cancer stem cell index and energy metabolic pathways were calculated (Figure 4C). As a result, PPP showed a strong positive correlation with the cancer stem cell index while glutaminolysis and glycolysis showed negative correlations with cancer stem cell index, and the correlation between FAO and the cancer stem cell index was insignificant. The relationship between PPP-related genes and the cancer stemness index was then verified using publicly published data. It was found that a G6PD-knockout prostate cancer cell line showed significantly lower CSI [22] (Figure 4D), while a G6PD-overexpression multiple myeloma cell line showed higher CSI [23] (Figure 4E). These results implied that upregulation of PPP-activity levels has a causal relationship with cancer stemness, at least in some cancer types.

### 3.5. HCC Cell Lines Mimic the Different Metabolic Patterns of Corresponding Human Tumors

**Figure 5:**
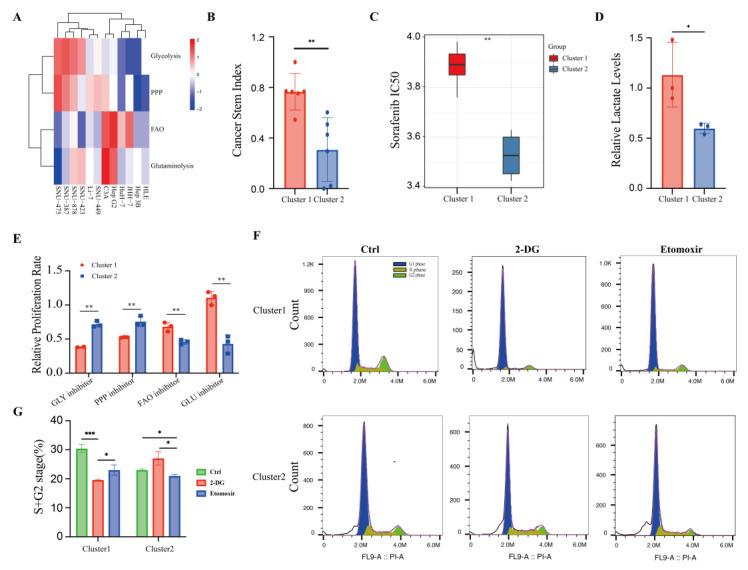
Liver cancer cell lines revealing potential metabolic therapy targets. (**A**) A heatmap showing the metabolic pathway activity levels of 12 common liver cancer cell lines from CCLE. (**B**) Cancer stem indexes of two clusters of liver cancer cells. (**C**) Boxplots of estimated IC50 values for sorafenib. (**D**) Relative lactate production of liver cancer cells. (**E**) Relative proliferation rate upon four metabolic perturbations in HCC cell lines. (**F**,**G**) Cell cycle progression upon two metabolic perturbations in HCC cell lines: up, SNU-449; down, Hep G2; left, Ctrl; middle, 2-DG; and right, Etomoxir. IC50, half the maximal inhibitory concentration. *, *p* value < 0.05; **, *p* value < 0.01; ***, *p* value < 0.001.

We re-analyzed the gene expression profiles of twelve common HCC cell lines to assess the robustness of this energy metabolic classification system. These cell lines were also classified into two clusters according to the metabolic classifier (Figure 5A). We found cluster 1 cancer cells exhibited much higher CSIs (Figure 5B) and a higher predicted IC50 of sorafenib (Figure 5C), which indicated more aggressive characteristics and a worse response to current anti-tumor therapy. SNU-449 and Hep G2 were then selected to represent cluster 1 and cluster 2, respectively, for further metabolic analysis. Lactate is the main downstream metabolite of glycolysis. So, we first measured the lactate levels of both cells to validate this classification. We discovered that SNU-449 produced significantly more lactate than Hep G2 (Figure 5D). q-PCR also validated this stratification (data not shown), where SNU-449 showed a higher transcriptional expression of HK1 (a glycolysis gene) and G6PD (a PPP gene), while Hep G2 exhibited a higher expression of CPT1A (an FAO gene) and GLUL (a glutaminolysis gene).

Targeting cancer metabolism has been shown to be a promising approach in cancer therapy [9,40]. Thus, we treated SNU-449 and Hep G2 cell lines with drugs that target different metabolic pathways. Of the four tested drugs, 2-DG (a glycolysis inhibitor) and DHEA (a PPP inhibitor) [41] showed a more anti-proliferative effect on SNU-449, while Etomoxir (an FAO inhibitor) [42] and Telaglenastat (a glutaminolysis inhibitor) [43] exerted a more anti-proliferative impact on Hep G2 (Figure 5E). Subsequently, a cell cycle analysis was performed to further confirm this result. As shown in Figure 5F,G, the glycolysis inhibitor 2DG showed a stronger inhibition ability on the proliferation of SNU449, while Etomoxir inhibited Hep G2 proliferation much more effectively. These data showed that the inherent metabolic features displayed by different cell lines can partially mimic the metabolism heterogeneity among different HCC patients, which provides insights into the evaluation of metabolic heterogeneity and the identification of personalized druggable pathways of HCC.

### 3.6. Distinct Immune Microenvironments between HCC Clusters

**Figure 6:**
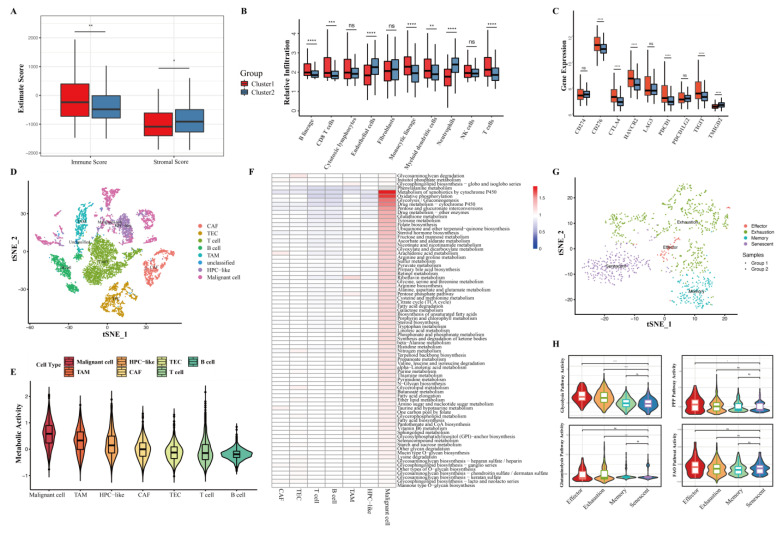
Distinct immune microenvironments between two liver cancer metabolic subtypes. (**A**) Immune and stomal scores calculated by the Estimate algorithm. (**B**) Relative abundance of immune and stromal cells. (**C**) Quantification of selected immune checkpoint genes in two clusters. (**D**) t-distributed stochastic neighbor embedding (t-SNE) plot of all cells from GEO: GSE125449. (**E**) Relative metabolic pathway activity levels among seven cell types from GEO: GSE125449. (**F**) Heatmap displaying KEGG metabolic pathway activity levels across seven cell types from GEO: GSE125449. (**G**) t-SNE plot of CD8+ T cells from GEO: GSE125449. (**H**) Violin plots depicting the four energy metabolic activity levels across different CD8+ T cell subtypes. ns, *p* value > 0.05; *, *p* value < 0.05; **, *p* value < 0.01; ***, *p* value < 0.001; ****, *p* value < 0.0001.

In order to explore the metabolic effects in the tumor immune microenvironment, the immune and stromal scores were calculated using the Estimate algorithm [29]. Interestingly, cluster 1 of the TCGA cohort showed a significantly higher immune score and a lower stromal score (Figure 6A). According to the cell populations counter (MCP-counter) algorithm (Figure 6B), cluster 1 showed an abundance of several immune cells, including B cells, T cells, and monocytic cells. To assess the functionality of these immune cells further, the expression of a number of immune checkpoint genes was compared between the two clusters (Figure 6C). Five of the six differentially expressed immune checkpoint genes were upregulated in cluster 1, including CTLA-4, PD-1, CD276, HAVCR2, and TIGIT. Furthermore, a significantly higher TIDE score was observed in cluster 1 (Supplementary Figure S3A), indicating a poorer response to immunotherapy. These results highlight that an exhausted immune microenvironment may partially contribute to the worse prognosis in cluster 1.

To better investigate the impact of the metabolic differences between the two clusters on immune cells, scRNA-seq data were retrieved from the GEO: GSE125449 database (Figure 6D). First, the overall metabolic activity levels among all the cells were estimated (unclassified cells were excluded) (Figure 6E). Unsurprisingly, malignant cells had the highest metabolic score, with most metabolic pathways upregulated (Figure 6F). Among all immune cells, those with immunosuppressive function (e.g., TAM) had higher levels of overall metabolic activity than those with anti-tumor function (e.g., T cells). Afterwards, the same unsupervised consensus clustering algorithm was used to classify all the malignant cells from 17 samples based on their energy and metabolic activities (Supplementary Figure S3B). The malignant cells were also robustly classified into two clusters with the same metabolic characteristics with respect to the bulk-seq data. The patients could also be assigned into two groups based on the cluster 1:cluster 2 ratio of the malignant cells. For example, Patient 16, who has 375 cluster 1 malignant cells and only 89 cluster 2 malignant cells, could be allocated to a cluster 1-like group. T cells were the most prevalent cells in the immune microenvironment regardless of groupings (Supplementary Figure S3C,D) and CD8^+^ T cells played a pivotal role in anti-tumor immunity. CD8^+^ T cells were further analyzed for metabolism effects and impacts between cells. CD8^+^ T cells could be classified into four clusters based on their transcriptional expression (Supplementary Figure S3D). It was found that most CD8^+^ T cells from the cluster 1-like group showed senescent characteristics (CD8-GZMK) [26], while the subtypes of CD8^+^ T cells from the cluster 2-like group were more diverse (Figure 6G). Interestingly, decreases in the glycolysis and PPP pathway activities were observed in senescent CD8^+^ T cells as compared to effector CD8^+^ T cells (Figure 6H). These phenomena indicated that metabolic competition between malignant cells and CD8^+^ T cells may lead to the alteration of the function of CD8^+^ T cells, resulting in an immunesuppressive environment.

## 4. Discussion

To sustain unconstrained growth, cancer cells undergo a very complex metabolic rearrangement, and thus their metabolic patterns vary enormously from those of fully differentiating cells [6]. Additionally, with the development of multiomics techniques, it has become clear that metabolic heterogeneity exists among tumors and even within distinct regions of solid tumors [7]. Based on three energy sources, we have established the first metabolic classifier for hepatocellular carcinoma. Independent cohorts have validated that HCC patients possess two kinds of energy metabolic patterns as follows: (1) cluster 1 with high glycolysis and PPP activity and (2) cluster 2 with high FAO and glutaminolysis activity. We thoroughly investigated the differences between the two clusters, including prognosis values, immune cell infiltration, oncogenic pathway activities, somatic mutation patterns, and cancer cell stemness. Multivariate Cox regression analysis and time-dependent ROC showed that the energy metabolic classifier was an independent and robust prognostic tool, indicating that metabolic heterogeneity in HCC should be taken into account in clinical practice.

With the intense investigation in the field of cancer metabolism since the discovery of the Warburg effect [44], it is now believed that abnormal metabolism plays a key role in the initiation and progression of cancer, rather than merely as a side effect of oncogenic rearrangements [45,46]. In this study, we found that cluster 1 showed higher oncogenic pathway activities, and the SNV analysis revealed that this difference was not a result of oncogene mutations. Of the ten oncogenic pathways in cluster 1, TP53 was the only one with a significantly higher mutation frequency. TP53 is the most frequently mutated gene in human tumors [47]. However, the exact mechanism of p53-dependent tumor suppression has not been fully elucidated. The P53 mutant protein may confer metabolic heterogeneity to cancer cells, thus endowing them with the ability to adapt to metabolic stress in the tumor microenvironment [48]. It was reported that p53 can inhibit glycolysis by downregulating the expression of glucose transporters [49] while inducing the expression of TP53-induced glycolysis and the apoptosis regulator (TIGAR) [50]. In addition, the p53 protein can regulate PPP activity by directly binding to glucose-6-phosphate dehydrogenase (G6PD) [51]. These results suggested that the high activity of glycolysis and PPP in cluster 1 could be partially explained by the TP53 mutation. However, developing p53-targeted therapy is not easy because the drug must specifically target mut-p53 in cancer cells while not affecting other cells harboring wt-p53 [52].

An increasing amount of evidence indicates that energy metabolism rearrangement is not just a consequence of lineage transition but a driving force in the maintenance of stemness and lineage commitment in human stem cells [53,54]. Additionally, some studies have shown that metabolic alteration contributes to the stemness of cancer cells [55,56]. In this study, we found that cluster 1 had a higher stemness index, which is strongly correlated with PPP activity. We further validated the correlation between PPP and cancer cell stemness in two independent cancer cell lines. Knockdown of G6PD decreased the stemness index, while overexpression of G6PD did the opposite. A possible explanation for this causal relationship is that it may be necessary to reprogram the energy metabolism of cancer cells to ensure that they are capable of meeting the metabolic stress of the tumor microenvironment. Huiyong Yin et al. proposed that hepatic aldolase B (Aldob) inhibits liver cancer tumorigenesis by directly binding G6PD, which is consistent with our analysis [57]. Thus, these results indicated that the blockage of specific genes in energy metabolic-related pathways, such as G6PD, could be a targetable therapeutic strategy to reverse the stemness phenotype of liver cancer cells.

Previous studies revealed that metabolism rearrangement can reshape the immune response in the liver, helping tumor cells evade immunosurveillance [17]. An estimated algorithm was used to calculate the immune and stromal scores. Cluster 1 showed significantly higher immune scores, which was validated by the MCP-counter algorithm. The tremendous success of immune checkpoint blockade [58] led us to further evaluate the function of these immune cells. Not surprisingly, the expression of several checkpoints, including PD-1 and CTLA-4, was significantly higher in cluster 1. We also found that the potential efficacy of immunotherapy in cluster 1 was worse as demonstrated by a higher TIDE score. This is consistent with previous studies suggesting that the high glycolytic activities of cancer cells may constrain glucose availability to tumor infiltration T cells, thereby inducing T cell exhaustion and immune evasion in HCC [59,60,61]. Finally, we increased the reliability of our study by exploiting a single-cell transcriptomics dataset of HCC. We found malignant cells are far more metabolically active when compared to non-malignant cells. Metabolic reprogramming increases the competitive strength of tumor cells to maintain unlimited proliferation in the tumor microenvironment (TME) with limited resources. Additionally, the unsupervised algorithm robustly classified the malignant cells into the same two clusters, which have the same characteristics with respect to patients. These results demonstrated that tumor cells were predominantly responsible for the metabolic heterogeneity in liver cancers. Cellular metabolism reprogramming is also essential for other proliferating immune cells, such as activated T cells [62]. Several studies have proposed that cancer metabolic reprogramming not only promotes tumorigenesis and cancer cells proliferating but also plays a role in the suppression of the antitumor immune response through several mechanisms, such as releasing immune-regulating metabolites, altering the expression of immune agents, and the suppression of antitumor immune cells [63]. In this study, we found most CD8^+^ T cells from cluster 1 displayed a senescent-like status (CD8-GZMK) [26] with low glycolysis and PPP activity levels. This phenomenon indicated that metabolic reprogramming does not only maintain the uncontrolled proliferation of malignant cells but also greatly impairs the anti-tumor capacity of immune cells by exerting metabolic stress on them [64]. Thus, this result reveals potential liver cancer therapies by combining metabolically targeted drugs and immune checkpoint inhibitors.

## 5. Conclusions

In conclusion, we established a novel metabolic classifier for disentangling the metabolic heterogeneity of hepatocellular carcinoma. Our integrative evaluation of this classifier indicated that metabolic reprogramming is related to clinical prognosis. The potential therapeutic significance of targeting unique metabolic dependencies was also revealed. However, further research is needed to overcome the two limitations of this study. First, only genomic and transcriptional levels of data were involved in the present study due to the lack of accessible proteomic and metabolomic datasets. Second, the complex cell-cell interactions and metabolism competition between malignant cells and immune cells should be investigated through more biological experiments in the future.

## Figures and Tables

**Table 1:** Clinical variables of the two clusters in the TCGA-LIHC datasets.

	Overall	Cluster 1	Cluster 2	*p*
N	363	133	230	
Gender = female/male (%)	118/245 (32.5/67.5)	48/85 (36.1/63.9)	70/160 (30.4/69.6)	0.321
Race (%)				0.062
American Indian or Alaska Native	1 (0.3)	0 (0.0)	1 (0.4)	
Asian	155 (42.7)	65 (48.9)	90 (39.1)	
Black or African American	17 (4.7)	9 (6.8)	8 (3.5)	
Not Reported	10 (2.8)	1 (0.8)	9 (3.9)	
White	180 (49.6)	58 (43.6)	122 (53.0)	
Age (median [IQR])	61.53 [51.97, 69.04]	61.09 [51.46, 68.29]	62.05 [52.78, 69.53]	0.446
AFP (median [IQR])	15.00 [4.00, 264.75]	79.00 [9.50, 3088.50]	8.00 [4.00, 47.00]	<0.001
Stage (%)				<0.001
Not reported	24 (6.6)	8 (6.0)	16 (7.0)	
Stage i	170 (46.8)	42 (31.6)	128 (55.7)	
Stage ii	84 (23.1)	38 (28.6)	46 (20.0)	
Stage iii	3 (0.8)	0 (0.0)	3 (1.3)	
Stage iiia	61 (16.8)	33 (24.8)	28 (12.2)	
Stage iiib	8 (2.2)	5 (3.8)	3 (1.3)	
Stage iiic	9 (2.5)	6 (4.5)	3 (1.3)	
Stage iv	1 (0.3)	0 (0.0)	1 (0.4)	
Stage iva	1 (0.3)	0 (0.0)	1 (0.4)	
Stage ivb	2 (0.6)	1 (0.8)	1 (0.4)	
T Stage (%)				<0.001
T1	180 (49.9)	43 (32.3)	137 (60.1)	
T2	89 (24.7)	42 (31.6)	47 (20.6)	
T2a	1 (0.3)	1 (0.8)	0 (0.0)	
T2b	1 (0.3)	1 (0.8)	0 (0.0)	
T3	42 (11.6)	22 (16.5)	20 (8.8)	
T3a	28 (7.8)	14 (10.5)	14 (6.1)	
T3b	6 (1.7)	2 (1.5)	4 (1.8)	
T4	13 (3.6)	8 (6.0)	5 (2.2)	
TX	1 (0.3)	0 (0.0)	1 (0.4)	
N Stage (%)				0.346
N0	246 (68.0)	95 (72.0)	151 (65.7)	
N1	4 (1.1)	2 (1.5)	2 (0.9)	
NX	112 (30.9)	35 (26.5)	77 (33.5)	
M Stage (%)				0.148
M0	262 (72.2)	104 (78.2)	158 (68.7)	
M1	3 (0.8)	1 (0.8)	2 (0.9)	
MX	98 (27.0)	28 (21.1)	70 (30.4)	
Tumor Weight (median [IQR])	150.00 [70.00, 315.00]	220.00 [110.00, 460.00]	110.00 [50.00, 250.00]	<0.001

## Data Availability

The original data presented in the study are presented in the article and supplementary materials, and other information will be available upon reasonable request to the corresponding authors.

## Supplementary Materials

The following supporting information can be downloaded at: https://www.mdpi.com/article/10.3390/cancers15030592/s1, Supplementary Table S1: Genes in four energy metabolic pathways. Supplementary Figure S1: Metabolic heterogeneity of liver cancer is associated with prognosis. (A) Heatmap showing four energy metabolic pathways activities of liver cancers in the ICGC cohorts. (B) Relative energy metabolic pathway activity of two clusters in the ICGC cohorts. (C) Kaplan–Meier curves of OS between clusters in the ICGC-LIHC datasets. (D) Time-dependent ROC curves of metabolic patterns in the TCGA datasets. (E) Boxplots for estimated IC50 values of sorafenib in the TCGA-LIHC datasets. Supplementary Figure S2: Distinct transcriptomic features between two liver cancer metabolic subtypes in the ICGC-LIHC datasets. (A) GSVA enrichment analysis of DEGs. (B) Quantification of 10 classical oncogenic pathways activity of two clusters. Supplementary Figure S3: Metabolic heterogeneity analysis based on single-cell gene expression. (A) TIDE scores between 2 clusters in TCGA-LIHC cohorts. (B) Heatmap displaying consensus clustering of metabolic patterns using the k-means algorithm (k = 2). (C,D) t-SNE plots of two groups from GEO: GSE125449. (E) Marker genes of four CD8+ T cell subgroups.
