# Supplementary material for: A High-Throughput Sequencing Data-Based Classifier Reveals the Metabolic Heterogeneity of Hepatocellular Carcinoma
Source: Cancers (Basel). 2023 Jan 18;15(3):592. doi: 10.3390/cancers15030592 (PMC9913608; doi:10.3390/cancers15030592)
Supplement: Supplementary file 1 [file cancers-15-00592-s001.zip › cancers-2092929-supplementary/Supplementary Figures S1-S3.pdf]

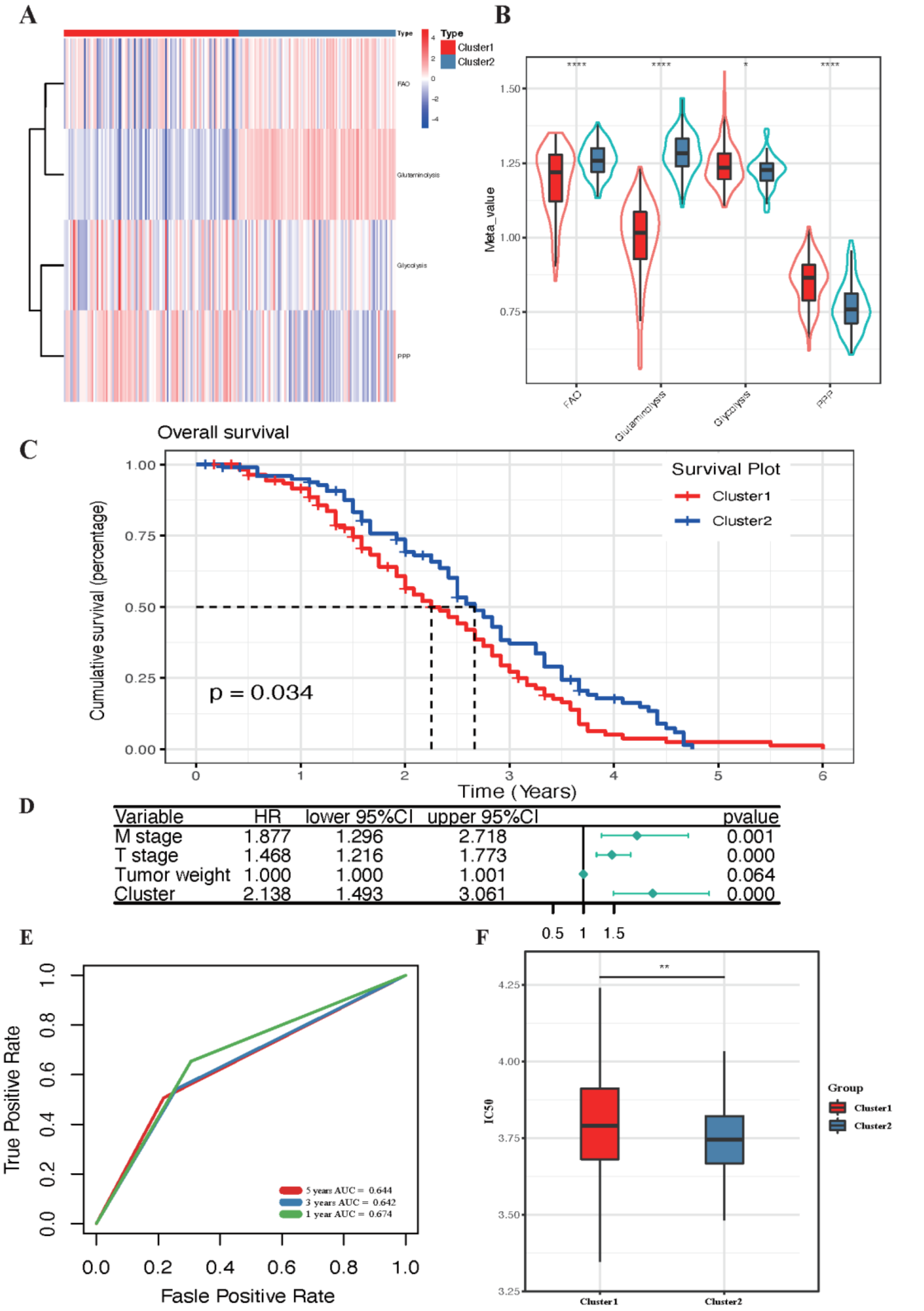

Supplementary Figure S1. Metabolic Heterogeneity of Liver Cancer is Associated with Prognosis. (A) Heatmap showing four energy metabolic pathways activities of liver cancers in the ICGC cohorts. (B) Relative energy metabolic pathway activity of two clusters in the ICGC cohorts. (C) Kaplan-Meier curves of OS between clusters in the ICGC-LIHC datasets. (D) time-dependent ROC curves of metabolic patterns in the TCGA datasets (E) Boxplots for estimated IC50 value of sorafenib in the TCGA-LIHC datasets.

A

B

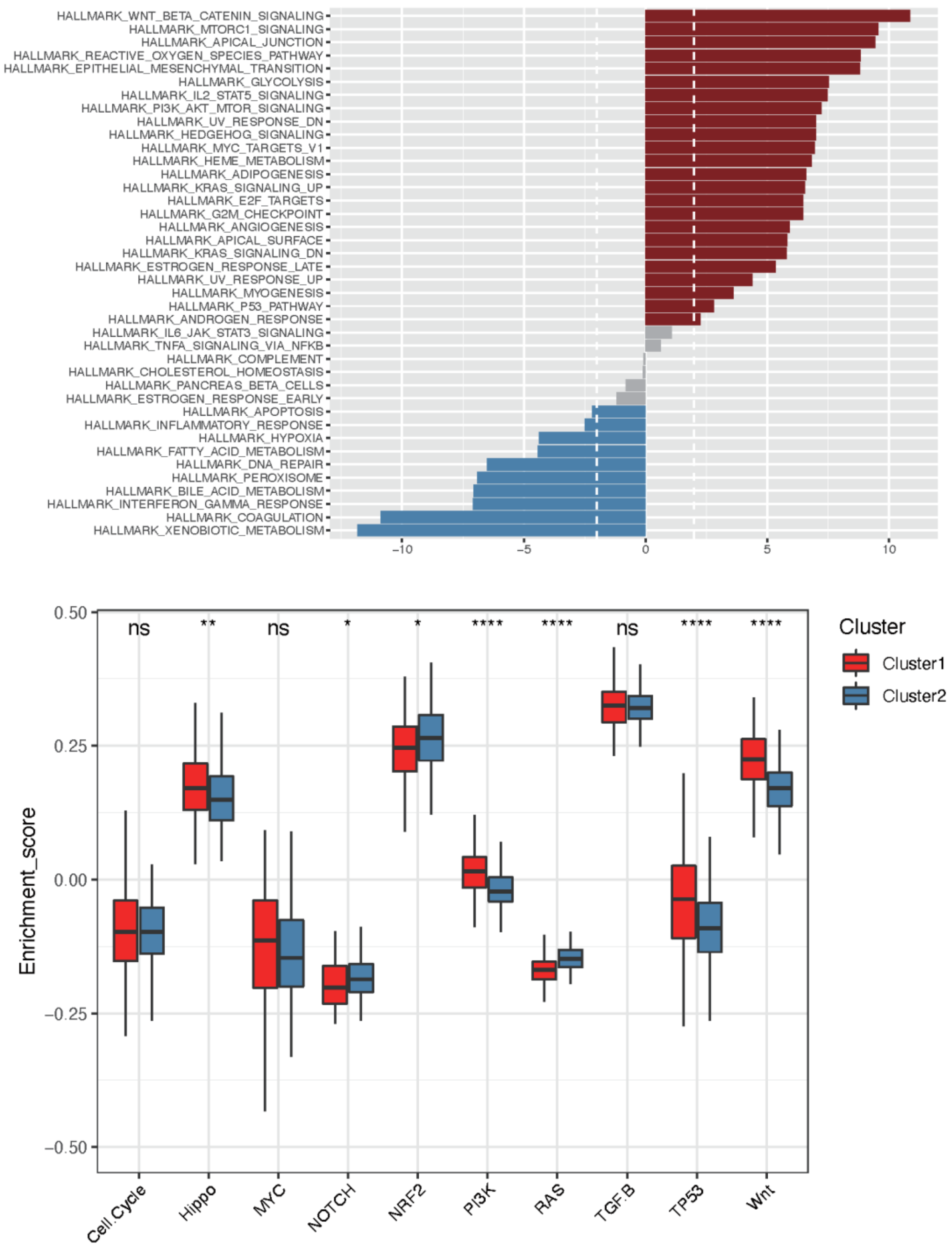

Supplementary Figure S2. Distinct Transcriptomic Features Be-tween Two Liver Cancer Metabolic Subtypes in the ICGC-LIHC sets. (A) GSVA enrichment analysis of DEGs. (B) Quantification of 10 classical oncogenic pathways activity of two clusters.

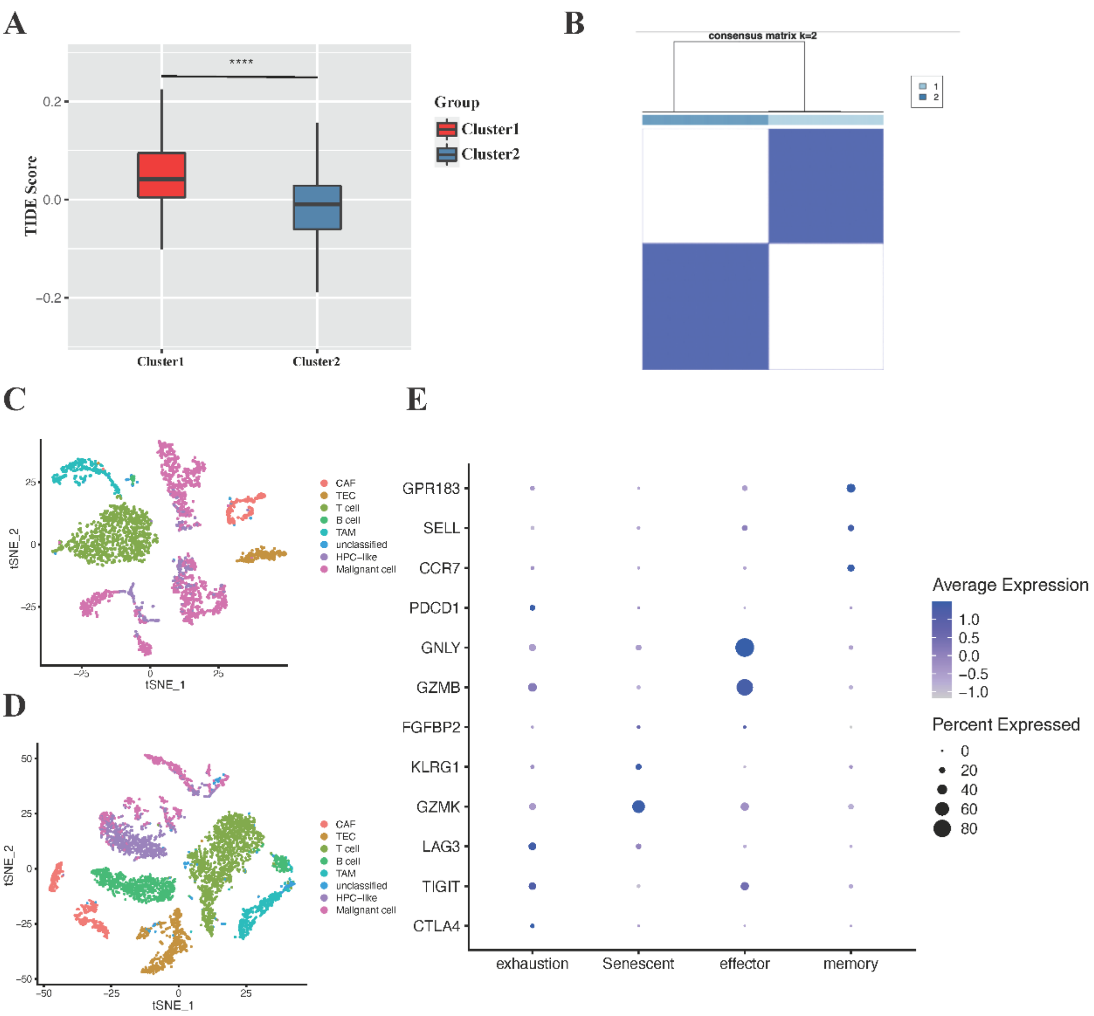

Supplementary Figure S3. Metabolic Heterogeneity Analysis Based on Single-cell Gene Expression. (A) TIDE scores between 2 clusters in TCGA-LIHC cohorts. (B) Heatmap displaying consensus clustering of metabolic patterns using the k-means algorithm (k = 2). (C-D) t-SNE plots of two groups from GEO: GSE125449. (E) Marker genes of four CD8+ T cell subgroups.
