# Supplementary material for: A High-Throughput Sequencing Data-Based Classifier Reveals the Metabolic Heterogeneity of Hepatocellular Carcinoma
Source: Cancers (Basel). 2023 Jan 18;15(3):592. doi: 10.3390/cancers15030592 (PMC9913608; doi:10.3390/cancers15030592)
Supplement: Supplementary file 1 [file cancers-15-00592-s001.zip › cancers-2092929-supplementary/Supplementary Table S1.pdf]

Supplementary Table S1. Genes in four energy metabolic pathways.

Glycolysis	PPP	FAO	Glutaminolysis
ENO1	G6PD	ACADM	GLS
ALDOA	H6PD	ACADVL	GLS2
GAPDH	PGLS	ACADS	GLUD1
PGK1	PGD	ACADL	GPT
SLC2A1	RPIA	ACAD9	GOT1
TPI1	RPE	ACADSB	GOT2
HK1	RPEL1	ACAD8	
GPI	TKT	HADHA	
PKM	TKTL1	HADHB	
PGAM1	TKTL2	HADH	
	TALDO1	ECHS1	
		EHHADH	
		ECI1	
		ECI2	
		HSD17B10	
		ACAA1	
		ACAA2	
		ACAT1	
		CPT1A	
		CPT1C	
		CPT2	
